# Surgical management of hallux valgus and hallux rigidus: an email survey among Swiss orthopaedic surgeons regarding their current practice

**DOI:** 10.1186/s12891-015-0751-7

**Published:** 2015-10-14

**Authors:** Lukas Daniel Iselin, Georg Klammer, Norman Espinoza, Panagiotis D. Symeonidis, David Iselin, Peter Stavrou

**Affiliations:** Department of Orthopaedics and Traumatology, University Hospital Basel, Spitalstrasse 21, CH-3041 Basel, Switzerland; Department of Orthopaedics and Traumatology, Kantonsspital Luzern, Switzerland; FussInstitut Zürich, Zürich, Switzerland; First Orthopaedic Department, Aristotle, University of Thessaloniki, “G. Papanikolaou” Hospital, Thessaloniki, Greece; KOF Konjunkturforschungsstelle, ETH Zürich, Zürich, Switzerland; Adelaide Orthosports Clinic, Adelaide, SA Australia

**Keywords:** Bunions, Foot surgery techniques, Forefoot, Toe, Midfoot, Survey

## Abstract

**Background:**

Various clinical and radiological criteria have been suggested to choose one of the numerous techniques in surgical treatment of hallux valgus and rigidus. We hypothesized that the surgeons' professional background will influence that choice depending on specialization, age, type and institution of training as well as his orthopaedic cultural orientation. Since Switzerland is characterized by regional languages (the most important being German and French), we were interested to learn if the linguistic differences had an influence on the orientation of the surgeons towards e.g. Anglo-American or French surgical traditions and/or sources of literature on the subject.

**Methods:**

A survey was e-mailed to all members of the Swiss Orthopaedic Society (SGOT-SSOT). Questions were asked regarding respondents’ demographics as well as their preferred treatment for 3 separate cases of (1) moderate and (2) severe hallux valgus and (3) hallux rigidus. The responses were collected and statistically analyzed.

**Results:**

Two hundred thirty of 322 respondents completed the survey(response rate 46 %). as they perform foot surgery on a regular base; 39 % were members of the Swiss Orthopaedic Foot and Ankle Society (SFAS). Selected surgical treatments differed as follows: in joint sparing procedures older and busier surgeons were more likely to use Chevron osteotomies, however more than 50 % preferred a Scarf-type of osteotomy. Along the so-called "Rösti-Graben" separating the French from the German speaking part of Switzerland no significant difference was found in the choice of operation technique.

Nevertheless the fact being a member of SFAS showed significant differences in technical choice in case 2 and 3.

**Conclusions:**

There are significant associations between the surgeons’ age, expertise and training and their preferred operative intervention. Considerable differences in the surgical management were found in the practice of the general orthopaedic surgeons 72 and the foot and ankle specialists. The cultural background and training is not mirroring the classical Swiss east west discrepancy. Despite the large number of surgical options available for hallux valgus, only a small number were preferred by the majority of surgeons.

**Electronic supplementary material:**

The online version of this article (doi:10.1186/s12891-015-0751-7) contains supplementary material, which is available to authorized users.

## Background

Hallux valgus and hallux rigidus are common conditions for which numerous operative interventions have been described in the literature [1–4]. Various clinical and radiological criteria have been used to guide the choice of surgical technique [5–7]. The surgeons’ professional background may influence that choice, depending on surgeons’ specialization, age, type and institution of training as well as their orthopaedic cultural orientation [8–20]. In a survey performed with the members of the Australian Orthopaedic Association the surgeon’s membership to the Australian Foot and Ankle Association and age influenced the choice of treatment most. Younger surgeons with a selective foot and ankle training tend to do more Scarf osteotomies in mild to moderate cases and metatarsaophalangeal (MTP)-I fusions in severe Hallux valgus or rigidus. Furthermore a trend to less joint replacements is visible [21].

Switzerland is divided into several distinct cultural and linguistic regions that were formed through the variable influences of the surrounding empires (French, German, Austrian and Italian) over time. An influence of the language difference on literature search, decision-making and practice due to membership of the surgeon in e.g. French or Anglo-American professional organization could not a priori be excluded.

Thus we presented the questions of the Australian survey to Swiss orthopaedic surgeons aiming to identify factors that influenced their choice of treatment with special emphasis on that demographic peculiarity [21].

## Methods

A survey was electronically mailed out to members of the Swiss Society of Orthopaedic Surgery and Traumatology (Schweizer Gesellschaft für Orthopädie und Traumatologie, SGOT) including fellows and registrars in the orthopaedic training program. Participants completed the survey questionary online via a dedicated website which collected and collated the responses. Translations into the three main languages (German, French and Italian) were available.

The first question of the survey was if the respondent performed foot and ankle surgery; a positive response allowed them to complete the remainder of the survey.

**Fig. 1:**
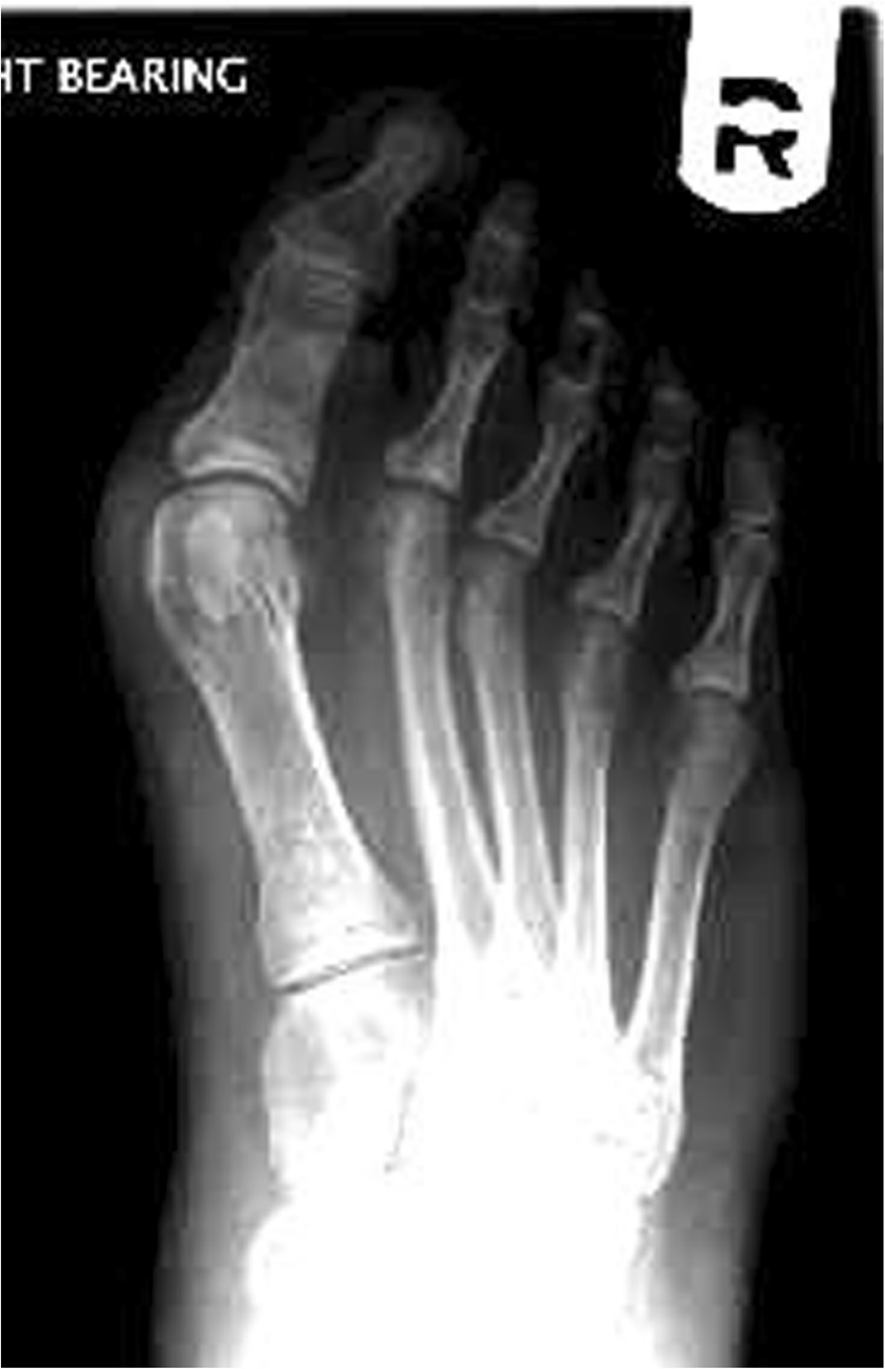
Survey case 1: Dorsoplantar weight-bearing radiograph of a patient’s right foot. History of complaints related to her hallux valgus deformity since one year, seeking surgical treatment after conservative measures had failed

**Fig. 2:**
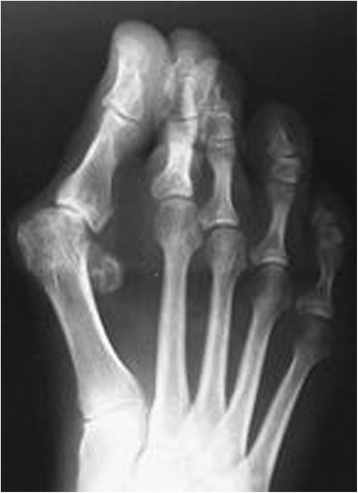
Survey Case 2: Dorsoplantar weight-bearing radiograph of a patient’s right foot. Complaints related to her hallux valgus deformity lasting since 2 years. She favours surgical treatment as conservative measures had failed

**Fig. 3:**
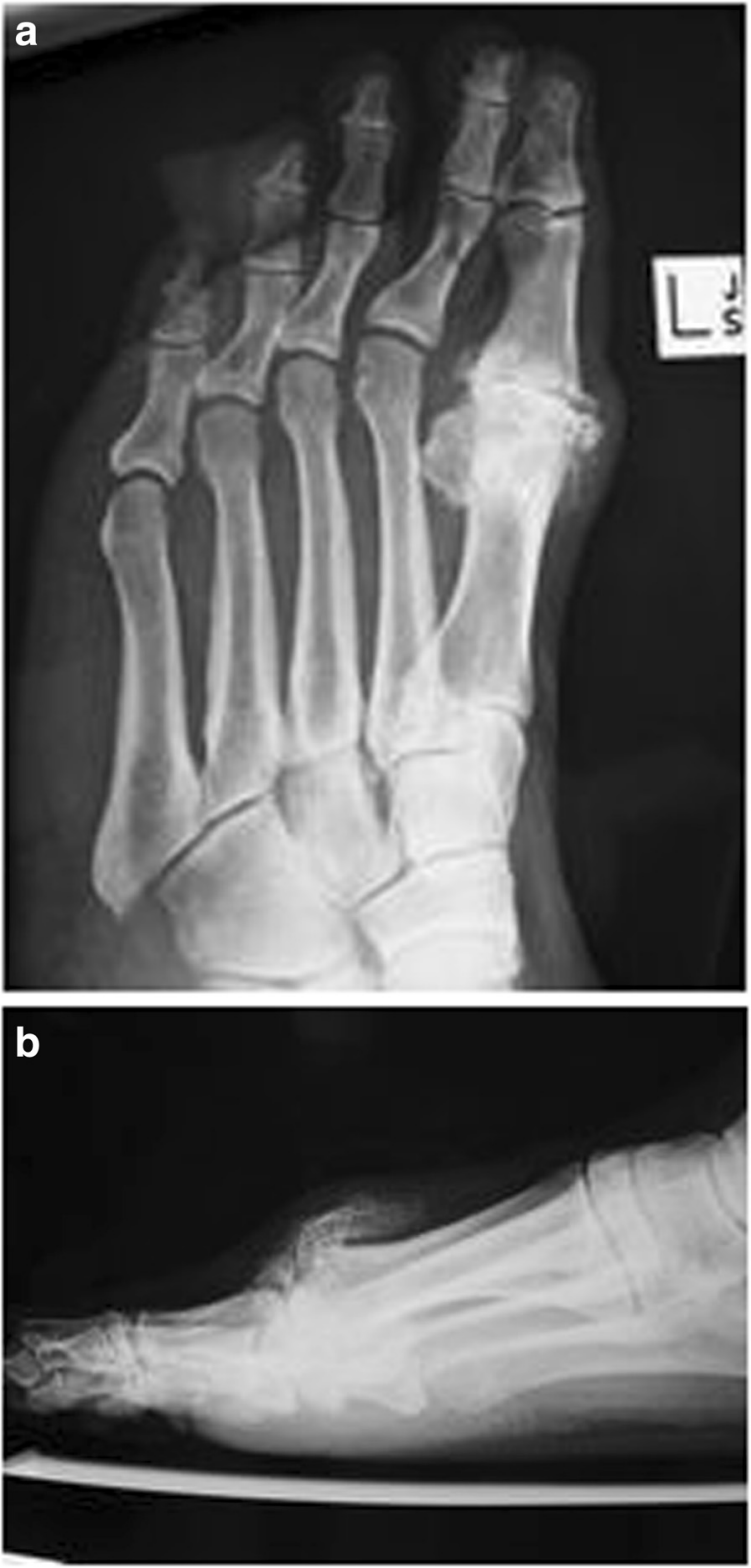
**a**, **b** Survey Case 3: Oblique and lateral views of a patients left foot. Painful hallux rigidus since one year. Conservative measures have failed and the patients seeks surgical treatment

Three separate cases were presented in the survey. Expecting a higher response rate the X-rays illustrating the cases were accompanied with only brief information on patient history in order to minimize the time needed for the completion of the survey. In Figs. 1, 2, 3 the X-rays with corresponding texts (translated in English) as given in the survey are depicted.

Case 1 (Fig. 1) described a patient with a moderate hallux valgus deformity (hallux valgus angle 34°; intermetatarsal angle 11°) and a congruent joint without evidence of joint space narrowing.

Case 2 (Fig. 2) showed a patient with a severe hallux valgus deformity with significant lateral displacement of the sesamoids (hallux valgus angle 44°, intermetatarsal angle 17°) an incongruent joint and some narrowing of the joint space.

Case 3 (Fig. 3a/b) illustrated a patient suffering of advanced degenerative osteoarthritis of the first metatarsophalangeal joint with significant dorsal osteophyte formation.

The questions to the cases asked the participant to state which surgical technique with what type of fixation they would choose and for cases 1–2 if an additional distal soft-tissue release would be performed. Answers were selected as multiple choice options including one for alternative solutions which the participant could state in detail (Table 1).

**Table 1:** Demographic Information

Survey recipients	654
Respondents	322/654 (=overall response rate 46 %)
Board certified orthopaedic surgeons	322/322 (100 %)
Trainees	0/322 (0 %)
Performing Foot & Ankle Surgery	230/322 (71 %) = response rate 35 %)
SFAS-Members^a^	90/322 (28 %)
Survey participants	230
Age groups (years of age)	
31–40	35/230 (15 %)
41–50	91/230 (39 %)
51–60	85/230 (38 %)
61–70	18/230 (8 %)
Case load (forefoot cases per year)	
0–10	8/230 (4 %)
< 25	58/230 (25 %)
25–50	71/230 (31 %)
> 50	93/230 (40 %)
Type of practice	
Public hospital	97/230 (43 %)
Private practice	104/230 (45 %)
University hospital	14/230 (6 %)
Other institution	13/230 (6 %)
Orthopaedic Training	
Fellowship	52/230 (22 %)
Orthopaedic Training Area	
German part	190/230 (82 %)
French part	24/230 (10 %)
Italian part	0/230 (0 %)
Mix German-French	12/230 (5 %)
Mix German-Italian	2/230 (1 %)
Mix french-Italian	2/230 (1 %)
Language/Region of Practice	
German-Speaking	183/230 (79 %)
French-Speaking	38/230 (17 %)
Italian-Speaking	5/230 (2 %)
Other	4/230 (2 %)

^a^SFAS = Swiss Foot & Ankle Society; 2 % did not report on membership

In order to correlate the chosen treatments to demographic data the following data was collected from each participant of the survey: number of surgical cases on the foot and ankle treated per year; most commonly used language (German, French, Italian, English); principal Swiss region of medical training (primary German speaking-, French speaking-, Italian speaking region or combinations thereof); type of institution of practice (University hospital; public hospital; private practice or mix of private and public); age and membership in the SFAS (Swiss Foot and Ankle Society).

The responses were collated and then organised into an appropriate format for transfer to a statistical programme, SAS version 9.2 (SAS Institute Inc, Cary, NC, USA). Statistical analysis was performed to obtain percentages of all of the responses and chi squared tests were undertaken to investigate for significant statistical relationships between responses and demographic variables.

There was no need for an ethical approval as the survey did not concern direct patient data according to our institutional review board (directed by the Head of the Orthopaedic Department of the University Hopital Basel, Prof M. Jakob) and the local ethical committee (EKNZ). The study was completed by medical professionals. See questionnaire here (Additional file 1).

## Results

The survey was mailed to 654 recipients according the list of members provided by the SGOT with an overall response rate of 46 % (322 responses). Seventy-one percent (230) of these stated to perform surgery on the foot and ankle, while 29 % did not (74).

So the response rate of the survey was actually 35 %. Demographic factors as well as participants case loads, type of training and practice are highlighted in Table 2. In summary all were board certified orthopaedic surgeons with forty percent of the respondents with a special interest in foot and ankle surgery and corresponding case loads.

**Table 2:** Results of the survey for procedure of choice for treatment in Case 1 (mild hallux valgus)

distal Chevron	95/230 (41 %)
Scarf	84/230 (36 %)
other	23/230 (10 %)
ReveL	9/230 (3.5 %)
prox Chevron	8/230 (3.5 %)
Lapidus (TMT-I fusion)	4/230 (2 %)
Ludloff	4/230 (2 %)
Keller’s procedure	2/230 (1 %)
Bunionectomy	1/230

For case 1 (moderate hallux valgus) distal Chevron was the most commonly chosen procedure (41 %). 78 % would perform a distal soft tissue (McBride) procedure in addition. Scarf osteotomy was the next most commonly chosen procedure (36 %). The correction was more likely to be accompanied by a McBride procedure 78 %) and was more likely to be preferred by members of SFAS (60 vs. 25 %, *p* <0.001). See details in Table 2.

In case two (severe hallux valgus) a Lapidus procedure was the most commonly preferred (31 %). It was more likely to be performed by members of the SFAS (53 vs. 26 %, *p* = 0.003) and by those who were less than 50 years old (47 vs. 12 %, *p* <0.001). 21 % preferred first metatarsophalangeal joint fusion with 80 % of those choosing a plate and screw construct for their fixation. See details in Table 3.

**Table 3:** Results of the survey for procedure of choice for treatment in Case 2 (severe hallux valgus)

Lapidus (TMT-I-fusion)	70/230 (31 %)
MTP-I-fusion	48/230 (21 %)
Scarf	38/230 (16 %)
Other	30/230 (13 %)
Ludloff	14/230 (6 %)
prox Chevron	13/230 (6 %)
distal Chevron	9/230 (4 %)
Keller’s procedure	6/230 (2 %)
none	2/230 (1 %)

Case 3 (first MTPJ arthritis), first MTPJ fusion was the treatment of choice for the majority of respondents (78 %) (Table 3) with 27 % preferring a plate and screw construct for fixation of the fusion and 60 % choosing screw fixation alone. Joint replacement arthroplasty was preferred by 2.2 % of respondents with a statistically significant percentage of them being in a practice which was 100 % private. Cheilectomy was chosen by 8 % and was more likely to be undertaken by those greater than 50 years old (12 vs. 1 %, *p* = 0.002). See details in Table 4.

**Table 4:** Results of the survey for procedure of choice for treatment in Case 3 (hallux rigidus)

MTP-I-fusion	177/230 (78 %)
Cheilectomy	20/230 (8 %)
Other	19/230 (8 %)
Joint replacement	5/230 (2 %)
Keller’s procedure	2/230 (1 %)
Interposition arthroplasty	2/230 (1 %)
none	1/230

The cultural background analysis did not show any differences regarding the choice of treatment in comparison with the language.

Further statistical analysis see Table 5.

**Table 5:** Statistical analysis by Pearson's Chi squared test

Comparison	*p*-value	Significant difference
Language vs Choice Case 1	*p*-value = 0.47	No
Language vs Choice Case 2	*p*-value = 0.28	No
Language vs Choice Case 3	*p*-value = 0.11	No
SFAS membership vs number of operations	*p*-value <0.001	Yes (SFAS members do operate more F&A cases than not members
SFAS membership vs Language background	*p*-value = 0.86	No differences
SFAS membership vs Choice Case 1	*p*-value = 0.89	No
SFAS membership vs Choice in Case 2	*p*-value < 0.001	Yes
SFAS membership vs Choice in Case 3	*p*-value = 0.014	Yes
Age and operations	*p*-value = 0.52	No differences in age and operations

## Discussion

We were able to obtain a large number of respondents from our target population, which constituted a representative sample with an appropriate mix of fellows of the Swiss Orthopaedic Association. The large numbers of respondents and inclusion of orthopaedic surgeons who are not foot and ankle specialists provided results which gave us a good overview of the current treatment practices for forefoot deformity surgery in Switzerland.

A weakness of the study is the low survey respond rate of only 35 % of all registered orthopaedic surgeons. On the other hand 71 % of the respondents (230 surgeons in a country with about 8 Mio inhabitants) were performing foot and ankle surgery on a regular base.

In a recent survey of academic American orthopaedic foot and ankle surgeons in mild bunion cases 87 % preferring a distal metatarsal osteotomy, followed by a more proximal osteotomy and in 10 % augmented by an additional Akin osteotomy [22].

Compared to the in 2012 published survey of Australian orthopaedic surgeons we found less parallels in age and training as well as geographic/cultural differences as expected [9].

It was interesting to note that the classical east–west differences in cultural and language in Switzerland is not correlated with the type of treatment chosen in forefoot surgery. Nevertheless being a member of the SFAS did appear to be a significant factor in the choice of the type of correction preferred in the more severe cases with a larger proportion of SFAS members choosing a Lapidus for case 2 or a MTP-I-joint fusion in the hallux rigidus over other type of operations [9].

The scarf osteotomy has been described as a more involved and complex osteotomy than other types (ie distal chevron) and as such may be more commonly performed by the surgeon who more regularly performs, or has a more dedicated interest in foot and ankle surgery such as members of the SFAS. Many argue the merits of the scarf osteotomy are that it can provide significant degree of correction with less risk of metatarsal head avascular necrosis and better healing because of the biomechanical stability of the osteotomy [23].

In case 1 it was noted that a proximal Chevron and a scarf osteotomy was associated with a higher rate of distal soft tissue procedure than the distal type of osteotomy. Fifty percent of the foot and ankle surgeons (F + A) performed a scarf while 52 % of the general surgeons did so.

In case 2 with the severe bunion over 50 % of the F + A surgeons but only a third of the general orthopaedic surgeons would have chosen a scarf osteotomy in combination with distal soft tissue releases equal distribution of around 80 %. A Lapidus procedure was the preferred method to fix this condition in 32 % of all SGOT members while only 3 % of the F + A specialists did choose this here. The main difference was actually seen in the comparison of the french swiss to the German speeking swiss orthopaedic surgeons. It is clearly seen that the Lapidus procedure is more often used in the french part than in the German speeking.

Another demographic variable which we found to be associated with differing choices of treatments was the age of the surgeon. We grouped people into either older or younger than 50 years of age. In case 1, a Mitchell osteotomy was more likely to be performed by those older than fifty. In case 2, a Keller’s excision arthroplasty was more likely to be performed by those surgeons older than fifty whilst in case 3, a Cheilectomy alone was more likely to be performed by those greater than 50 years of age. These three findings were interesting as they may represent a change not only in the teaching and training of orthopaedic surgeons in Switzerland over the past years but possibly also a change in the concepts of aetiology and patho-physiology of hallux valgus as a condition.

Joint replacement arthroplasty was not a very common choice for the management of case 3 (hallux rigidus) in 2 % of the F + A surgeons and 5 % of the general orthopaedic surgeons, with most respondents preferring to perform a first MTPJ fusion.

With all of the available treatments described in the literature for hallux valgus it was interesting to note in our study that there were only a few procedures which were preferred by the majority of the swiss orthopaedic surgeons. Five procedures (Scarf, distal chevron, proximal chevron, 1st MTPJ fusion and 1st MTPJ replacement) accounted for the major percentage of preferred treatments for each of the three case examples [6, 8, 12, 13, 15, 18, 24].

The other interesting finding was the significant differences in the practise of general orthopaedic surgeons and F + A specialists. The F + A specialists tend to perform more modern surgeries such as the scarf osteotomy with additional distal soft tissue releases and usually don’t perform arthroplasties in the highly loaded first MTP joint.

## Conclusion

There are significant associations between the surgeons’ age, expertise and training and their preferred operative intervention. Considerable differences were found in the practice of the general orthopaedic surgeons and the foot and ankle specialists. The cultural background and training is not demonstrating the expected classical Swiss east–west discrepancy. Despite the large number of surgical options available for hallux valgus, only a small number were preferred by the majority of surgeons. While we are all anecdotally aware that lesser deformity is treated with distal osteotomies and more severe deformity with a proximal osteotomy, we are aware of only limited data in the literature that verifies this. We could show differences in the swiss orthopaedic population in regard to the membership of the specialist’s society but the “Rösti-Graben” seems not to be as deep as mostly seen in the cultural and social correlation.

## Additional file

Additional file 1:
**Appendix: Questionnaire.** (DOCX 32 kb)

## Abbreviations

SGOT: Schweizer Gesellschaft für Orthopädie und Traumatologie/Swiss Society of Orthopaedic Surgery and Tramatology
SFAS: Swiss foot and ankle society
