# Supplementary material for: Surgical management of hallux valgus and hallux rigidus: an email survey among Swiss orthopaedic surgeons regarding their current practice
Source: BMC Musculoskelet Disord. 2015 Oct 14;16:292. doi: 10.1186/s12891-015-0751-7 (PMC4607006; doi:10.1186/s12891-015-0751-7)
Supplement: Additional file 1: — Appendix: Questionnaire. (DOCX 32 kb) [file 12891_2015_751_MOESM1_ESM.docx]

**Survey questions to cases 1-3**

| **Questions to cases 1 and 2** |
| --- |
| 1. Which surgical technique would you choose?   **Ο** Bunionectomy  **Ο** Resection Arthroplasty (Keller’s)  **Ο** Distal Chevron-Osteotomy  **Ο** Ludloff  **Ο** Proximal Chevron-Osteotomy  **Ο** Scarf  **Ο** Lapidus (TMT-I Arthrodesis)  **Ο** other ______________________________   1. Which type of fixation would you use?   **Ο** none  **Ο** K-wire(s)  **Ο** Screw(s)  **Ο** Clip(s)  **Ο** other ______________________________   1. Would you perform an additional distal soft-tissue release (McBride)?   **Ο** yes  **Ο** no |
| **Questions to case 3** |
| 1. Which surgical technique would you choose?   **Ο** Cheilectomy  **Ο** Resection Arthroplasty (Keller’s)  **Ο** Interpositional arthroplasty  **Ο** artificial joint replacement (total/hemi-arthroplasty)  **Ο** MP-I-Arthrodesis  **Ο** other ______________________________   1. Which type of fixation would you use?   **Ο** none  **Ο** K-wire(s)  **Ο** Screw(s)  **Ο** Clip(s)  **Ο** Plate and Screws |
